# The Medicinal Treatment of Chlorosis

**Published:** 1901-01-19

**Authors:** 


### THE MEDICINAL TREATMENT OF CHLOROSIS.

In a lecture delivered at the West London Hospital, Dr. Seymour Taylor [1] dealt more especially with the use of iron in the treatment of chlorosis. This metal, he says, is practically regarded as a specific. Notwithstanding this, unsatisfactory results of treatment by it are not infrequent, while it constantly happens that a patient under treatment by one doctor with no good results turns to another and obtains relief. Yet iron is in the prescriptions of both. The fact is that other things besides iron are required. The old if somewhat vulgar simile, attributed to a St. Bartholomew's physician, by which a chlorotic girl is compared to cast linen, is probably correct. He is credited with saying that these patients require "washing out, ironing, and then airing," and certain it is that every preparation of iron has its value in the treatment of chlorosis considerably enhanced by a preliminary "washing out" by a course of purgative medicine. Iron, again, should be taken so as to be digested along with the food. The amount of iron in hæmoglobin is infinitely small, and probably the quantity contained in a single bottle of ordinary ferruginous medicine would amply supply all the deficiency if only it were digested. The difficulty is to get it absorbed, and this is best done by so timing the dose that the iron shall, as it were, enter the system on a vehicle of albumen.

One may summarise the conclusions arrived at in regard to the treatment of chlorosis by iron by saying—first, that the carbonate is the most efficacious remedy; secondly, that whatever preparation of iron be prescribed it must be fresh and free from oxidation; thirdly, that until the intestinal canal is clear and the digestive functions are in good order no preparation of iron is of much or any avail. There are, however, other considerations to be remembered. In the dyspeptic form of chlorosis special attention to the digestive organs may be necessary for some time, several weeks, before iron is given; the neurotic form may border upon the anorexia nervosa described by Gull, and in such cases valerian may be very useful; while in the plump but very pallid and breathless type arsenic may be given in addition to the iron. In any case, however, one must, week by week, estimate the percentage of hæmoglobin in the blood. If this shows even a small improvement the prescription may be continued, but if the amount of hæmoglobin remains stationary or diminishes another preparation of iron must be found. To get to the root of chlorosis, however, other things besides mere physic have to be considered. But that is another question.

[1] Clin. Journ., Jan. 2.

